# Supplementary material for: A stochastic numerical approach for a class of singular singularly perturbed system
Source: PLoS One. 2022 Nov 28;17(11):e0277291. doi: 10.1371/journal.pone.0277291 (PMC9704572; doi:10.1371/journal.pone.0277291)
Supplement: Attachment — Submitted filename: Authors Response R2 28-07-2022.docx [file pone.0277291.s002.docx]

**Author Response**

**Paper Title:**

A stochastic numerical approach for a class of singular singularly perturbed system

by

Zulqurnain Sabir, Thongchai Botmart, Muhammad Asif Zahoor Raja, Wajaree weera, Fevzi Erdoğan

**ID: PONE-D-21-38900R1**

**Journal: PLOS ONE**

Reviewers' comments:

Reviewer #1: All comments have been addressed

**Authors reply**

Many thanks for your valuable remarks and encouraging recommendations

Reviewer #2: All comments have been addressed

**Authors reply**

Many thanks for your valuable remarks and encouraging recommendations

Many thanks for the

Editorial Comments

Thank you for submitting your manuscript to PLOS ONE. After careful consideration, we feel that it has merit but does not fully meet PLOS ONE’s publication criteria as it currently stands. Therefore, we invite you to submit a revised version of the manuscript that addresses the points raised during the review process.

Two reviewers have completed the review of your manuscript. Although they suggested acceptance, during our in-house check, **we noticed that your manuscript contains significant references of other works by the same authors. PLOS ONE stresses that the literature review and reference list should accurately reflect the state of the field, and opposes excessive citations. Please check and revise your manuscript again following this criteria, and submit a revised version. We cannot rule out the possibility of inviting a third reviewer.**

**Authors reply**

First of all many thanks for both reviewer for acceptance of our manuscript in your esteemed journal after address the technical comments.

We have updated the manuscript after addressing the editorial remarks regarding the reference cited in the literature review,; accordingly, after detailed, careful and critical review of the literature review for the justification of the problem statement, we have cited only those references accurately reflect/portray the state of the field. Additionally we have avoided the extensive citation of the references of the particular author as suggested for better understanding and readability.

Please see the introduction and reference section of the revised manuscript.
